# Challenges of blockchain adoption for manufacturing supply chain to achieve sustainability: A case of rubber industry

**DOI:** 10.1016/j.heliyon.2024.e39448

**Published:** 2024-10-17

**Authors:** Alok Yadav, Anish Sachdeva, Rajiv Kumar Garg, Karishma M. Qureshi, Bhavesh G. Mewada, Muhammad Musa Al-Qahtani, Mohamed Rafik Noor Mohamed Qureshi

**Affiliations:** a Department of Industrial and Production Engineering, Dr. B. R. Ambedkar National Institute of Technology, Jalandhar, 144008, India; b Department of Mechanical Engineering, Parul Institute of Technology, Parul University, Waghodia, 391760, India; c Department of Industrial Engineering, College of Engineering, King Khalid University, Saudi Arabia, Abha, 61421, Saudi Arabia

**Keywords:** Supply chain, Blockchain, Industry 4.0, Manufacturing, Sustainability, Challenges, CoCoSo, Fuzzy DEMATEL

## Abstract

Global market competitiveness and standards are pushing the manufacturing supply chain to adopt sustainable approaches to enhance quality, reduce waste, improve environmental practices, and optimize product costs. Blockchain technology has emerged as a promising tool for improving supply chain transparency and manufacturing sustainability. However, various challenges to the mainstream use of blockchain must be identified and addressed. In this regard, the study aims to identify and comprehend the challenges of adopting blockchain technology in the manufacturing sector to improve sustainable standards. To bridge this gap, the study used a three-step procedure. First, through a comprehensive literature review, 19 potential challenges were identified. Then, with the experts' discussion, a total of 18 challenges were finalized. In the second phase, data was collected from 15 professionals with industrial backgrounds. Lastly, the collected data was analyzed using Combined Compromise Solution methodologies and the Fuzzy-Decision-Making Trial and Evaluation Laboratory (fuzzy DEMATEL). This hybrid methodology prioritizes the challenges in blockchain adoption and categorizes them into causal groups. This improves the decision-making process by evaluating how the mutual impact of challenges in blockchain adoption affects achieving sustainability in the manufacturing supply chain. The outcomes of the presented work are validated through a sensitivity analysis of the outranked dimensions of blockchain adoption challenges. Results show that 'High Initial Investment Cost' and 'Lack of Digital Skills’ are the top challenges in its adoption. The findings of this work provide a roadmap to make the manufacturing supply chain sustainable and accomplish sustainability.

## 1 Introduction

In the era of globalization, the increased population and individual demands heavily depend on the manufacturing supply chain that consumes a significant amount of natural resources [1,2]. The complexity of managing and controlling manufacturing supply chains to achieve sustainability has increased due to globalization. Regulatory organizations and demand from customers are pressuring the manufacturing industry to adopt digital technology strategies into their business models to solve environmental issues and attain sustainability [3].

Currently, manufacturing industries across the globe that consume substantial global natural resources face challenges with greenhouse gas emissions, which pose key issues related to tackling sustainability [4]. In the context of sustainability issues, advanced technology is a key factor in transforming the supply chain management scenario [5]. Supply chain operations can be improved through the integration of advanced technologies, which have significant consequences for environmental sustainability [6]. As supply chains attempt to evaluate sustainable practices and demand visibility between their stages continuously, the issues become more complicated. A growing number of stages, suppliers, and marketplaces have brought attention to concerns about visibility related to tracing, identification, and accessibility [7]. To meet individual demands, the manufacturing industry is simultaneously going through a digital transition called Industry 4.0, which incorporates various digital technologies like automation, blockchain, IoT, and artificial intelligence into the manufacturing process [8]. The combination of these technologies (for instance, automation and the Internet of Things) supports decentralized decision-making processes, customized production, efficient energy utilization, contemporaneous operation tracking and oversight, and predictive maintenance for manufacturing industries [9].

In the past decade, advanced technological concepts have been linked with the United Nation's sustainable development goal to enable the attainment of manufacturer sustainability [10,11]. The phases involved in the manufacturing supply chain must be closely monitored. This is mainly because an individual unsustainable process may cause the entire process as a whole to become unsustainable [12]. Therefore, a blockchain-based inter-organizational information-sharing method is essential for systematically evaluating sustainable supply chain practices. Previous studies [13,14] indicate that blockchain can open new possibilities for sustainability. Blockchain technology provides a robust framework for enhancing sustainability in manufacturing SC through its core features of transparency, traceability, and efficiency [15]. By ensuring that all stages of the SC are transparent, blockchain fosters accountability and builds consumer trust in sustainable practices. Its traceability feature provides high quality and compliance with environmental and safety regulations, while its efficiency reduces administrative burdens and the carbon footprint of its operations [16]. Moreover, blockchain's capability to promote and verify sustainable practices ensures manufacturers can meet global standards and consumer expectations for sustainability.

The comprehensive integration of blockchain can lead to significant environmental benefits, such as reduced waste, lower carbon emissions, and more efficient use of resources. It also supports social sustainability by ensuring ethical practices and fair labour conditions. As manufacturers increasingly adopt blockchain technology, it will play a key role in transforming the industry towards a more sustainable and responsible future.

The idea and application of blockchain technology are still in their initial stages, and new concepts for developing countries like India and need a comprehensive understanding awareness of business procedures [17]. With the manufacturing sector accounting for fifteen percent of GDP and twelve percent of employment, India is one of the developing economies with the fastest growth rates in the world [5]. The Network Readiness Index (2023), which places India 60th out of 134 nations, highlights that India still lags economies like China in deploying advanced technologies. The degree of industrial automation in India's manufacturing sector is still low compared to other industrialized nations, especially in the rubber, leather, textile, pharmaceutical, and automotive industries. Using blockchain technology, the Indian government plans to increase employment in the manufacturing sector and improve the overall economy [18].

To position India as a hub for global advanced manufacturing, aligning the ‘Make in India’ campaign with the blockchain concept is imperative. Nonetheless, the industry is still at an early stage of implementation [19]. To achieve the sustainability target, industries must prioritize sustainability goals simultaneously. To fulfil India's ambition of becoming a hub for innovative manufacturing and promoting supply chain sustainability, its adoption in the manufacturing sector faces substantial challenges that need to be overcome.

Blockchain technology can potentially revolutionize the manufacturing supply chain by enhancing transparency, traceability, and efficiency. Its applications range from tracking material provenance to automating complex logistics processes. Despite its promise, blockchain adoption in the Indian manufacturing sector is still in its early stages and faces significant challenges.

This study aims to explore the key challenges to blockchain adoption in the Indian manufacturing industry, focusing on the rubber industry. By identifying and prioritizing these challenges, this research provides a roadmap for overcoming them and promoting sustainable practices. The present study addresses the following research questions (RQs).

RQ1 What are the challenges in adopting blockchain technology for a sustainable manufacturing supply chain?

RQ2 How can these challenges be prioritized in the context of the Indian rubber industry?

RQ3 What are the cause-and-effect relationships among these challenges?

To accomplish these objectives, the present study uses a questionnaire-based survey of the Indian rubber industry. A survey of the Indian rubber industry was conducted. This study used a comprehensive literature review to find the challenges in adopting Blockchain technology that affects the sustainability of the manufacturing supply chain. Further, in the decision-making process, a hybrid approach of Combined Compromise Solution (CoCoSo) and fuzzy-decision-making trial and evaluation laboratory (fuzzy DEMATEL) is used to rank the identified challenges and cause-and-effect relations among the challenges, respectively.

The remainder of the paper is organized as follows: Section 2 presents the literature review. In Section 3, we explore the research methodology. Data analysis and results are discussed in Section 4. The discussion of the outcomes is found in Section 5. The conclusions, implications, limitations, and future research scope of the study are discussed in Section 6.

## 2 Literature review

To identify the challenges, research gaps, and opportunities for Blockchain technology adoption, a comprehensive literature review is carried out. The literature review is integral to the research process, providing a comprehensive understanding of the complexities within manufacturing supply chain dynamics. This understanding shapes the research objectives, highlighting the need to address key challenges that can promote its adoption for achieving sustainability. The impact of blockchain at various stages of the manufacturing supply chain has been studied.

It is essential to address these challenges to achieve sustainability. In this context, technology acts as a catalyst for transforming current supply chain practices. The literature review demonstrates the potential of blockchain to enhance transparency, traceability, and efficiency. The study of mutual interdependencies of these challenges concerning sustainability goals facilitates the decision-making process. By analyzing the research literature, the neglected aspects of these challenges can be explored concerning the dynamics of the manufacturing supply chain.

### 2.1 Industry 4.0 and blockchain

**Fig. 1:**
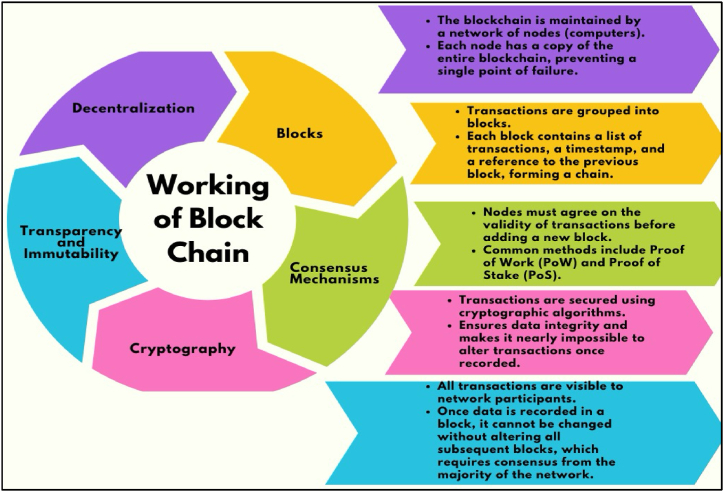
Blockchain and working procedure (Authors' work).

In the era of globalization, the resilience of any industry across the globe mainly depends on market competitiveness. Nowadays, businesses are driven to identify their customers using sustainability variables such as environmental, social, and economic factors, especially by adopting I4.0 technology, including Blockchain. Blockchain has the potential to process valuable information in different areas [20]. The I4.0 concept in the manufacturing industry drives blockchain technology [21]. Blockchain is a “disruptive technology that is revolutionizing information technology and represents a change of cultural paradigm for how information is shared [14]”. The working procedure of blockchain technology is represented in Fig. 1.

The customer demands must be focused on and met for customer satisfaction. I4.0 technology such as blockchain has forced the manufacturing industry to revisit and integrate its business model [22]. Due to increased market competitiveness, the Indian manufacturing industry is moving towards the I4.0 in its business model for technological advantages. I4.0 helps combine physical and advanced technology and provides more flexibility in the manufacturing supply chain to achieve sustainability. Table 1 summarizes past studies conducted on blockchain and sustainability.

**Table 1:** Existing studies in blockchain and sustainability with contribution.

Author	Blockchain	Sustainability	Industry sector	Manufacturing perspective	Method	Contributions
[23]	✓	✓	×	✓	SLR	Explore how blockchain can enhance sustainability in manufacturing supply chain.
[24]	✓	✓	✓	×	SLR	Analyze the main barriers to blockchain adoption in supply chain.
[25]	✓	✓	×	×	SLR	Investigate the role of blockchain in increasing transparency and traceability.
[26]	✓	✓	×	×	SLR	Analyze the financial challenges of adopting blockchain technology.
[13]	✓	✓	×	×	SLR	Examine the impact of digital skills on blockchain adoption in manufacturing.
[27]	✓	✓	✓	×	Survey	Evaluate policy frameworks supporting blockchain for sustainability.
[17]	✓	✓	✓	×	Survey	Assess the potential of blockchain to reduce carbon footprints in supply chains
[14]	✓	✓	×	✓	Survey	Provide empirical insights into factors influencing blockchain adoption.
[28]	✓	✓	×	×	SLR	Develop a theoretical framework for understanding blockchain's impact.
[29]	✓	✓	×	×	SLR	Analyze how blockchain can enhance supply chain resilience.
[2]	✓	✓	×	×	SLR	Identify key challenges in implementing blockchain in the supply chain
[7]	✓	✓	✓	×	SLR	Investigate the use of blockchain and smart contracts supply chain
[13]	✓	✓	×	×	SLR	Evaluate blockchain's potential to achieve sustainable development goals.
[24]	✓	✓	×	×	SLR	Explore the role of blockchain in improving supply chain visibility
[30]	✓	✓	×	×	SLR	Bibliometric analysis of blockchain and manufacturing industry.

### 2.2 Manufacturing supply chain and blockchain technology

The adoption rate of blockchain in the Indian manufacturing industry has risen because of its crucial role in minimizing waste, detailed tracking from extraction to finished goods, increased transparency, ethical sourcing, energy efficiency, and supporting the circular economy [1]. In this context, the existing literature has discussed blockchain technology's potential benefits in traceability, energy efficiency, waste reduction, transparency, and support for the circular economy [3]. Despite the many advantages of blockchain technology, the previous studies in this field show a lack of understanding of the concept of blockchain in the manufacturing industry, due to blockchain knowledge constraints [2,3].

Due to government pressure and the dynamic customer market, the manufacturing industry in emerging economies is recognizing the need for blockchain technology to improve resource efficiency, decentralized decision-making, and energy management [3]. Blockchain and other I4.0 technologies support collaborative environments in supply chain networks, facilitating sustainability across them. Therefore, it is crucial to identify its adoption challenges in the manufacturing industry [30]. In this context, we have identified the critical challenges of blockchain adoption in the Indian rubber industry, as represented in Table 2.

**Table 2:** Blockchain adoption challenges in manufacturing supply chain.

Challenge	Description	References
Integration of Technology (C1)	Challenges in merging blockchain with existing systems	[13,31]
Lack of R&D (C2)	Poor effort in research and development	[32]
Resistance to Change (C3)	Facing problems in easy adoption	[19]
High Initial Investment Cost (C4)	Large upfront expenses for blockchain implementation	[19,33]
Maintenance cost (C5)	High costs are required in maintenance	[34]
Supplier Support (C6)	Inadequate support from suppliers and partners	[25]
Lack of Digital Skills (C7)	Employees are not well-trained digitally and have fewer skills	[35]
Lack of Awareness (C8)	Low understanding and knowledge of blockchain	[26,36]
Lack of Infrastructure (C9)	Insufficient technological infrastructure	[3,37]
Lack of Support from Government Policies (C10)	Lack of support from government policies regulations and incentives	[38]
Data Security and Privacy Issues (C11)	Confidential data protection and maintaining privacy	[39]
Data Collection and Management (C12)	Facing challenges in the management of large amounts of data	[40]
Restricted Laws and Regulations (C13)	Limiting legal frameworks hindering blockchain use	[41]
Organizational Requirement and Readiness (C14)	Readiness and alignment of organizational structures	[42]
Existing Job Disruption (C15)	Potential negative impact on current jobs	[43,44]
Reliability and Stability (C16)	Ensuring consistent and stable blockchain performance	[9]
High Complexity (C17)	Complexity in understanding and implementing blockchain solutions	[19,45]
Lack of Expertise (C18)	Deficiency of skilled professionals in blockchain technology	[19,46]

### 2.3 Research gaps

Based on insights from the core research literature, the following research gaps have been identified.

1. Past studies are concentrated on a theoretical understanding of the integration of Blockchain technology and the manufacturing supply chain and lack empirical investigation in the context of the emerging economy, particularly in India, which requires further investigation.
2. The challenges of introducing blockchain remain unresearched; therefore, a study that leads to causal relationships regarding challenges of introduction must be further investigated

To address these gaps, hybrid decision-making approaches and comprehensive surveys are necessary.

### 2.4 Framework development

**Fig. 2:**
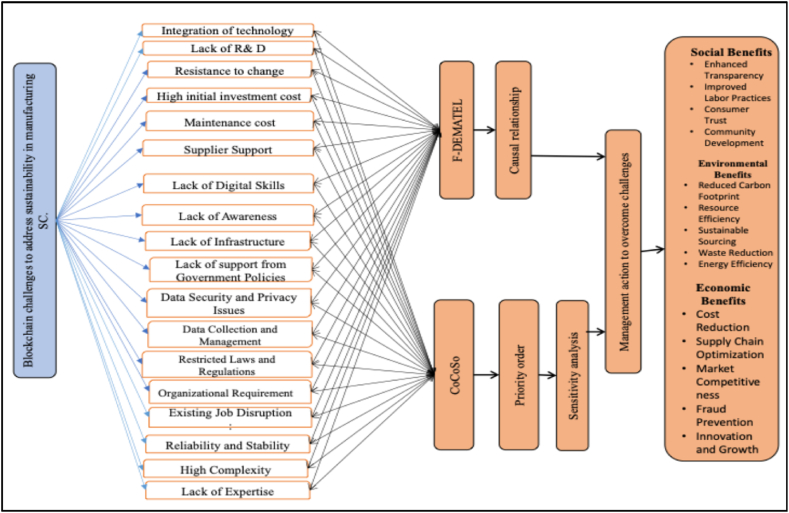
A developed framework of Blockchain challenges in manufacturing supply chain (Authors' work).

Based on our literature review, a theoretical framework for Blockchain adoption for a sustainable manufacturing supply chain has been developed and is depicted in Fig. 2.

## 3 Research methodology

**Fig. 3:**
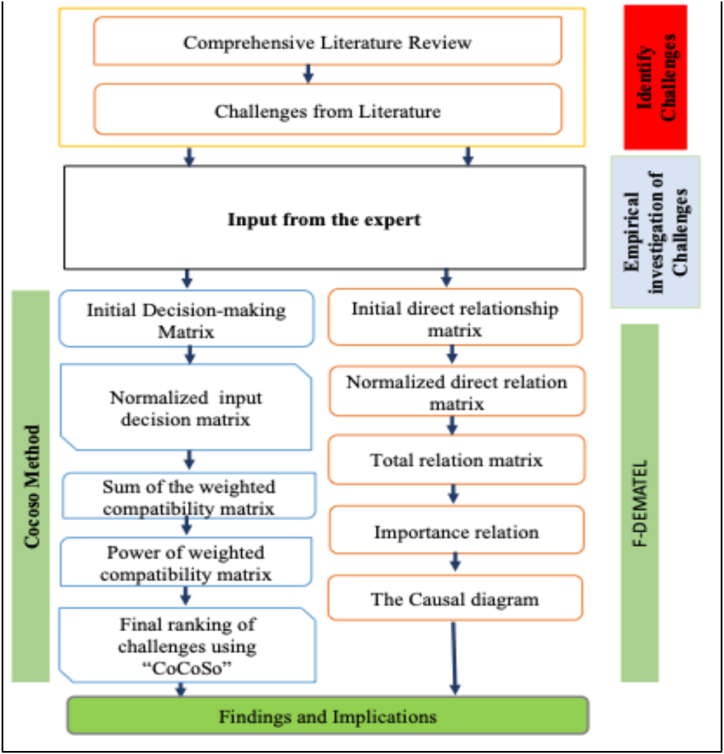
Research methodology used in this study.

The study adopts a hybrid approach, incorporating a comprehensive literature review, expert opinions, and inputs from fifteen experts in the rubber industry during the questionnaire design process. The details of the research methodology are represented in Fig. 3. A brainstorming session with fifteen experts was conducted within the Indian manufacturing sector to validate the 18 identified challenges that can support the adoption of blockchain in promoting a manufacturing supply chain.

### 3.1 Questionnaire design and data collection

The study targeted the Indian manufacturing sector (Rubber industry), aiming to validate identified challenges as outlined in the study's objectives. Initially, a questionnaire survey using a 1–5 Likert scale was developed and validated through a pilot study to refine the questions. Based on expert feedback, a few modifications were made to the questionnaire according to the inclusion criteria. The final questionnaire included a formal cover letter explaining the data collection objectives and a consent form. After collecting the data, the responses were cleaned and tested for validity and reliability. The data were collected over a period from February 20, 2024, to March 31, 2024.

The initial list of identified blockchain adoption challenges was presented to the expert panel, which asked them to indicate which practices they deemed relevant for industries in the manufacturing sector (rubber industry). After collecting their responses, a final list of 18 challenges was agreed upon following further discussion within the group. Once the blockchain challenges were finalized, they were prioritized using the CoCoSo method.

This study is conducted in three distinct phases aligned with the research objectives. In the first phase, blockchain adoption challenges are identified through an extensive literature review. These challenges are then discussed with a panel of 15 experts to complete the list, focusing on the context of emerging economies. In the second phase, we ranked the finalized challenges based on their importance using the CoCoSo, a recently developed and efficient multi-criteria decision-making (MCDM) method. In the third phase, we employed the fuzzy DEMATEL to analyze causal relationships among the challenges.

### 3.2 Why CoCoSo and fuzzy DEMATEL?

Analytic hierarchy process, Analytic Network Process, Best Worst Method etc., are the most adopted multi-criteria decision-making techniques that can prioritize challenges. The hierarchy complexity in AHP and the response complexity in BWM are less suitable. AHP works best in linear hierarchical systems whereas ANP may be adopted in the case of complex hierarchy. The CoCoSo approach is independent of *criteria weights* and provides solutions consistent with other MCDM approaches [45,46]. Additionally, it can discriminate between optimal alternatives with a greater resolution [47]. It has been employed because of its increased stability and dependability. The fuzzy DEMATEL approach improves traditional DEMATEL. Fuzzy logic can handle uncertainty and vagueness in expert judgments to provide precise modelling, greater flexibility with input data, and better decision-making.

## 4 Data analysis and results

To validate the feasibility of the developed framework, practitioners from manufacturing supply chain units in Northern India were consulted, and brainstorming sessions were conducted. Experts provided insights on various challenges under consideration and explored their practical implications. A questionnaire with rating points was developed, and evaluations were obtained from field experts. The inclusive criteria adopted were experts from the manufacturing domain with a minimum experience of five years. Experts having more than five years of experience, ranging from six to twenty years of working with manufacturing supply chain and related operations were involved. The senior-level engineering positions varied from section executive to section head, and in managerial positions ranging from assistant manager to general manager. The age range was from 36 to 53 years. A section head was the only female expert. Based on the responses received from the experts, it was further checked for reliability. The Cronbach alpha coefficient was found to be 0.837, indicating that the data is consistent and reliable for analysis. All the challenges related to blockchain adoption for a manufacturing supply chain had a mean range between 2.87 and 3.87, whereas the standard deviation varied from 0.96 to 1.49. The CoCoSo technique involves a linguistic scale shown in Table 3, facilitating respondents to provide feedback based on the significance of the challenges [5].

**Table 3:** The linguistic scale used for CoCoSo.

Linguistic terms	Response
No Influence	1
Low Influence	2
Medium Influence	3
High Influence	4
Max Influence	5

### 4.1 Combined Compromise Solution

The CoCoSo is based on the combination of simple additive weighting and an exponentially weighted product model. It deals with the ranking or selection of alternatives. Furthermore, they are assessed against specific criteria. The steps of the CoCoSo are provided in Ref. [18].

Using these steps, the challenges are ranked according to the decreasing value of *Ki*, meaning that a higher value of *Ki* is more important. The initial decision-making matrix for the CoCoSo method is shown in Table 4.

**Table 4:** Initial decision-making matrix.

Challenges	EX 1	EX 2	EX 3	EX 4	EX 5	EX 6	EX 7	EX 8	EX 9	EX 10	EX 11	EX 12	EX 13	EX 14	EX 15
C1	2	3	5	3	2	2	2	3	3	3	5	4	2	4	2
C2	2	2	3	4	4	2	3	3	4	4	3	5	5	4	3
C3	5	3	5	1	4	2	2	2	1	4	5	2	2	3	5
C4	4	2	5	2	5	2	4	2	3	2	4	2	3	5	4
C5	5	4	5	3	4	4	5	5	5	2	3	5	3	2	3
C6	3	2	2	3	4	3	3	5	2	5	4	3	2	2	3
C7	3	1	2	5	5	2	2	3	5	3	4	3	3	5	3
C8	5	2	2	5	3	5	3	2	5	3	1	3	2	3	4
C9	5	2	2	2	5	3	1	2	5	5	5	2	3	4	4
C10	5	5	3	4	5	4	3	5	4	4	2	3	5	4	2
C11	4	4	4	4	4	3	3	2	3	3	2	4	2	5	2
C12	2	3	3	5	3	2	3	5	5	4	5	4	3	5	3
C13	5	5	3	3	1	3	3	3	4	2	2	2	2	1	4
C14	3	4	4	2	5	4	3	1	3	4	5	5	2	2	2
C15	4	2	2	1	4	3	4	4	5	1	3	4	5	2	3
C16	5	4	5	2	5	4	2	3	2	3	2	4	3	4	2
C17	2	5	5	5	4	5	2	4	2	3	2	3	5	2	1
C18	2	3	4	5	3	3	3	3	3	5	2	3	2	5	5

Normalising of the initial decision-making matrix is carried out. The expert's opinion is considered equally important, with a weight of 0.06. Various challenges are considered non-beneficial criteria. The normalized input decision matrix, the sum of the weighted compatibility matrix and the power of the weighted compatibility matrix, can be prepared. Based on the three aggregating methods, each challenge's relative weights (*Kia*, *Kib*, and *Kic*) are calculated and shown in Table 5.

**Table 5:** Final ranking of challenges using CoCoSo and aggregation.

Challenges	*Pi* + *Si*	*Ka*	Rank	*Kb*	Rank	*Kc*	Rank	*K*	Final Rank
C1	10.836	0.055	10	2.505	11	0.790	10	1.595	**10**
C2	9.846	0.050	16	2.276	16	0.718	16	1.450	**16**
C3	10.080	0.051	13	2.611	10	0.735	13	1.595	**11**
C4	13.680	0.070	1	3.072	1	0.998	1	1.978	**1**
C5	11.740	0.060	5	2.671	6	0.856	5	1.711	**5**
C6	10.820	0.055	11	2.503	12	0.789	11	1.594	**12**
C7	11.896	0.061	3	2.843	3	0.868	3	1.948	**2**
C8	10.999	0.056	6	2.728	5	0.802	6	1.693	**6**
C9	10.010	0.051	14	2.500	13	0.730	14	1.547	**13**
C10	11.848	0.060	4	2.787	4	0.864	4	1.763	**4**
C11	10.928	0.056	8	2.618	8	0.797	8	1.645	**8**
C12	10.689	0.055	12	2.334	15	0.780	12	1.519	**14**
C13	10.938	0.056	7	2.670	7	0.798	7	1.666	**7**
C14	12.885	0.066	2	3.072	2	0.940	2	1.934	**3**
C15	10.884	0.056	9	2.612	9	0.794	9	1.641	**9**
C16	8.130	0.041	18	2.158	18	0.593	18	1.307	**18**
C17	9.786	0.050	17	2.218	17	0.714	17	1.423	**17**
C18	9.924	0.051	15	2.388	14	0.724	15	1.498	**15**

### 4.2 Sensitivity analysis

**Fig. 4:**
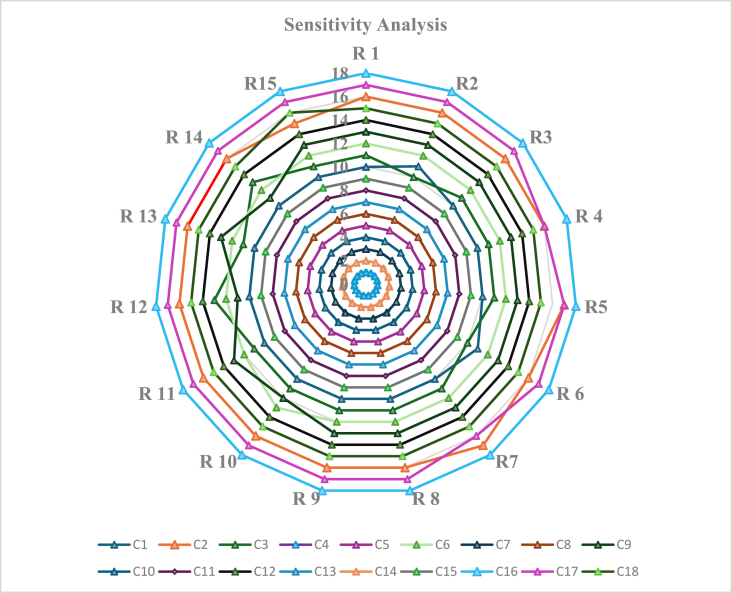
**S**ensitivity analysis for BCT Challenges' rank.

The robustness of the results is examined in this study through a sensitivity analysis. Using sensitivity analysis, decision-makers can understand how different scores and weight configurations affect the order in which challenges are prioritized. Experts' responses could be biased because of the disparity in their industry experience. We altered the weight of experts in an individual run to conduct the sensitivity analysis. We used sensitivity analysis to look at instances where certain variables changed in importance depending on input changes. Table 1 (Refer to Appendix A) presents the findings from the sensitivity analysis. It displays the associated ranks for each challenge and includes fifteen distinct runs created by varying the weights of each expert. It also shows that practically all challenges have the same or slightly different ranks. In 15 runs, the C4 challenge has the highest ranking, whereas the C16 task has the lowest ranking. Therefore, concluding that “High initial investment cost” is the most significant barrier to all the stated challenges is reasonable. This further demonstrates the sufficient reliability and stability of the technique selected to achieve the desired outcome. Fig. 4 represents the order of each run's Blockchain technology challenges.

### 4.3 Fuzzy DEMATEL method

A Fuzzy DEMATEL method is an MCDM or comprehensive technique for identifying and evaluating the causal relationship between various complex criteria. This technique helps to show how the criteria directly relate to one another and to determine how much each interacts. It has been suggested that it is appropriate for determining the overall influence strength across all criteria and the direct and indirect causal relationships. The inner dependencies between the several criteria are efficiently managed using this technique.

#### 4.3.1 The analysis procedure of fuzzy DEMATEL

The steps involved in the analysis procedures of fuzzy DEMATEL are well documented in Ref. [19]. Table 6 is used for decision-making. Table 2 is prepared for the Initial direct relationship matrix, whereas Table 3 is prepared for the total relation matrix, as shown in Appendix A. Based on these tables. Table 7 is prepared which shows the cause-and-effect relation.

**Table 6:** Linguistic scale for fuzzy DEMATEL.

Response	Linguistic terms	Triangular Fuzzy Number (TFN)
1	No Influence	(0.0, 0.1, 0.3)
2	Low Influence	(0.1, 0.3, 0.5)
3	Medium Influence	(0.3, 0.5, 0.7)
4	High Influence	(0.5, 0.7, 0.9)
5	Max Influence	(0.7, 0.9, 1.0)

**Table 7:** The cause-and-effect relation.

Challenges	*Ri*	*Ci*	*R*_*i*_ + *C*_*i*_	*R*_*i*_*-C*_*i*_	Cause/Effect	(*R*_*i*_ + *C*_*i*_*)/*2	Ti/AVERAGE OF *T*_*i*_
C1	1.864	1.328	3.192	0.536	Cause	1.596	0.188
C2	1.460	1.829	3.289	−0.369	Effect	1.644	0.194
C3	1.686	1.845	3.531	−0.159	Effect	1.765	0.208
C4	1.875	1.378	3.253	0.497	Cause	1.627	0.192
C5	1.598	2.102	3.701	−0.504	Effect	1.850	0.218
C6	1.476	1.675	3.151	−0.199	Effect	1.576	0.186
C7	1.335	1.721	3.056	−0.386	Effect	1.528	0.180
C8	1.625	1.623	3.248	0.002	Cause	1.624	0.191
C9	1.466	2.093	3.559	−0.626	Effect	1.780	0.210
C10	1.640	1.953	3.593	−0.313	Effect	1.796	0.212
C11	1.852	1.760	3.612	0.092	Cause	1.806	0.213
C12	1.864	2.069	3.933	−0.205	Effect	1.967	0.232
C13	1.779	1.811	3.590	−0.033	Effect	1.795	0.212
C14	1.767	2.083	3.850	−0.316	Effect	1.925	0.227
C15	1.380	2.095	3.475	−0.715	Effect	1.738	0.205
C16	1.763	2.046	3.809	−0.283	Effect	1.905	0.225
C17	1.541	2.418	3.959	−0.877	Effect	1.979	0.233
C18	1.772	1.868	3.640	−0.097	Effect	1.820	0.215

**Fig. 5:**
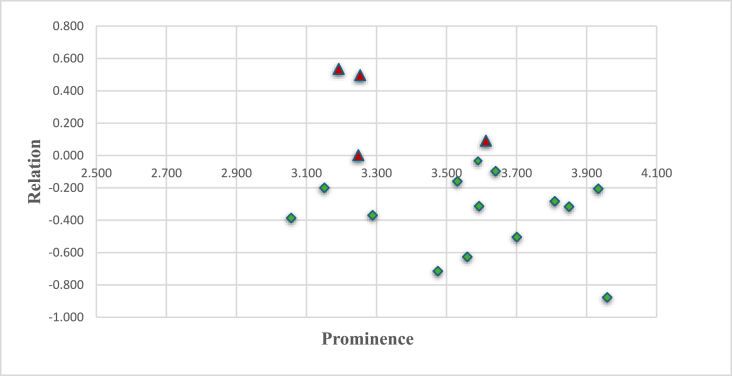
Causal diagram.

Fig. 5 shows the causal diagram with a clear overview of the implicit behaviour of the cause-and-effect groups. A red triangle represents elements in the cause group, while those in the effect group are shown in a green rectangle.

## 5 Discussion

To achieve the research objectives outlined in the introduction section, we conducted a comprehensive literature review to identify the critical challenges of blockchain adoption in the manufacturing supply chain. A specific manufacturing industry (the rubber industry) was selected for study. The analysis of the developed framework for blockchain adoption challenges in the manufacturing supply chain was conducted using a hybrid decision support system incorporating CoCoSo and fuzzy DEMATEL, which was used to assess the influence and causal interrelationships among the challenges.

The overall preference scores (Pi) using the CoCoSo approach for each challenge are obtained. It can be inferred that the overall preference score ranks the challenges. Based on this ranking, ‘High Initial Investment Cost(C4)’ is ranked highest and secures the top position. This is followed by the ‘lack of digital skills (C7)’ and ‘organizational requirements and readiness (C14)’. Reliability and Stability (C16) are the least significant challenges. The outcomes of this study from the cause-and-effect diagram reveal that the challenges, namely C1, C4, C8, and C11, belong to the cause group, while the remaining challenges belong to the effect group. Because challenges that belong to cause groups affect the whole system, their performance can affect the goal.

As a result, the challenges belonging to the cause group should get more attention. Among all the challenges belonging to the cause group, C1 has the highest score, which is 0.536, this means C1 contributes a greater impact on the whole system in adopting blockchain for the green supply chain. It means that C1 receives more attention.

This outcome aligns with past studies [19,25,48], that identified high costs and lack of expertise as major challenges. However, this study extends the literature by providing a detailed cause-and-effect analysis, revealing the interconnectedness of these challenges. We compare the existing DEMATEL methodology, along with the proposed hybrid approach, showing that the hybrid approach provides better outcomes [49].

Thus, the contributions of this study are significant from a theoretical and practical perspective.

## 6 Conclusion, implication, limitations and future research scope

The present study aims to identify and analyze the challenges of blockchain adoption in the manufacturing supply chain. Initially, nineteen challenges were identified, which were then refined to eighteen through experts' decisions. These eighteen challenges were further analyzed using a hybrid approach of the CoCoSo and the fuzzy DEMATEL methodology. A five-point linguistic rating scale was used and validated, and the robustness of this hybrid approach was confirmed through sensitivity analysis. The existing DEMATEL methodology with the proposed hybrid approach shows that the hybrid approach provides better outcomes.

The present hybrid method effectively prioritizes the challenges based on the *K* score. It quantifies the cause-and-effect relationship between the challenges, categorizing them into cause-and-effect groups. The CoCoSo analysis reveals that ‘High Initial Investment Cost (C4)’ is the foremost challenge in Blockchain technology adoption followed by the ‘lack of digital skills (C7)’ and ‘organizational requirements and readiness (C14)’. The practising managers must realize the importance of a sound and viable financial plan in Blockchain technology adoption. In the present study Reliability and Stability (C16) are found to be the least significant challenges hence, practicing managers may postpone their policy to attain it before other significant challenges. Fuzzy DEMATEL analysis reveals that the challenges, namely ‘Integration of Technology (C1)’, ‘High Initial Investment Cost (C4)’, ‘Lack of Awareness (C8)’, and ‘Data Security and Privacy Issues (C11)’, belong to the cause group. While the remaining challenges belong to the effect group. Among all the challenges belonging to the cause group, Integration of Technology (C1) has the highest score, which is 0.536, which means C1 contributes a more significant impact on the whole system in adopting Blockchain for the manufacturing supply chain. It implies that C1 gets more attention.

This research sheds light on the Blockchain technology challenges faced by the practising managers of the manufacture of supply chains. Analysing the case industry problem, it has been revealed that practising managers should focus on mitigating the risk of managing the investment plan. Digital skills pose the challenges hence employee skills may be enhanced through regular training. Managers should prioritize decision policies that favour innovation in production processes and supply chain practices, offering cost-effective technical solutions to real-time problems. The Blockchain technology adoption framework is designed to assist practitioners in implementing sustainable practices in the future.

The presented work can be extended to safeguard blockchain adoption interests in the manufacturing supply chain and achieve sustainability goals. Various regional studies could explore the potential of blockchain technology, facilitating the exchange of effective measures that promote responsible production and consumption mechanisms. Its feasibility can be explored to enhance transparency and effectiveness in sustainability practices.

This study identifies eighteen challenges to blockchain technology adoption in the manufacturing supply chain for achieving sustainability. Future research could include additional challenges depending on the blockchain technology adoption challenges. While this study is conducted in the Indian context, the proposed framework could be applied to other emerging economies for comparative analysis. Different decision-making approaches like DEMATEL, with an analytic network process, could also be explored. It may be able to capture the relationship beyond the linear hierarchy. Apart from sensitivity analysis, the effect of challenges may be modelled and validated using partial least squares structural equation modelling.

## CRediT authorship contribution statement

**Alok Yadav:** Software, Methodology, Data curation, Conceptualization. **Anish Sachdeva:** Investigation, Formal analysis, Data curation, Conceptualization. **Rajiv Kumar Garg:** Investigation, Formal analysis, Data curation, Conceptualization. **Karishma M. Qureshi:** Validation, Methodology, Formal analysis, Conceptualization. **Bhavesh G. Mewada:** Investigation, Formal analysis, Data curation, Conceptualization. **Muhammad Musa Al-Qahtani:** Software, Methodology, Data curation, Conceptualization. **Mohamed Rafik Noor Mohamed Qureshi:** Resources, Methodology, Investigation, Conceptualization.

## Informed consent statement

Not Applicable.

## Institutional review board statement

Not Applicable.

## Data availability

Data will be made available to researchers at their request.

## Funding

This research was funded by the 10.13039/501100023674Deanship of Scientific Research, King Khalid University, Kingdom of Saudi Arabia, and the grant number is RGP 2/476/44.

## Declaration of competing interest

The authors declare that they have no known competing financial interests or personal relationships that could have appeared to influence the work reported in this paper.

## Figures and Tables

**Table 1:** Outcomes from sensitivity analysis.

Challenges	R 1	R2	R3	R 4	R5	R 6	R7	R 8	R 9	R 10	R 11	R 12	R 13	R 14	R15
C1	10	11	10	10	10	11	10	10	10	10	10	10	10	10	10
C2	16	16	16	16	17	16	17	16	16	16	16	16	16	16	15
C3	11	10	11	11	11	10	11	11	11	11	11	13	11	13	11
C4	1	1	1	1	1	1	1	1	1	1	1	1	1	1	1
C5	5	5	5	5	5	5	5	5	5	5	5	5	5	5	5
C6	12	12	12	12	12	12	12	12	12	13	12	12	12	12	12
C7	3	3	3	3	3	3	3	3	3	3	3	3	3	3	3
C8	6	6	6	6	6	6	6	6	6	6	6	6	6	6	6
C9	13	13	13	13	13	13	13	13	13	12	13	11	13	11	13
C10	4	4	4	4	4	4	4	4	4	4	4	4	4	4	4
C11	8	8	8	8	8	8	8	8	8	8	8	8	8	8	8
C12	14	14	14	14	14	14	14	14	14	14	14	14	14	14	14
C13	7	7	7	7	7	7	7	7	7	7	7	7	7	7	7
C14	2	2	2	2	2	2	2	2	2	2	2	2	2	2	2
C15	9	9	9	9	9	9	9	9	9	9	9	9	9	9	9
C16	18	18	18	18	18	18	18	18	18	18	18	18	18	18	18
C17	17	17	17	16	17	17	16	17	17	17	17	17	17	17	17
C18	15	15	15	15	15	15	15	15	15	15	15	15	15	15	16

**Table 2:** Initial direct relationship matrix.

Challenges	C1	C2	C3	C4	C5	C6	C7	C8	C9	C10	C11	C12	C13	C14	C15	C16	C17	C18	SUM
**C1**	0.743	0.676	0.300	0.100	0.300	0.185	0.765	0.576	0.765	0.185	0.185	0.576	0.576	0.378	0.765	0.576	0.576	0.576	8.805
**C2**	0.020	0.743	0.100	0.500	0.500	0.185	0.378	0.185	0.185	0.378	0.185	0.576	0.185	0.378	0.765	0.576	0.765	0.185	6.791
**C3**	0.300	0.676	0.743	0.100	0.100	0.576	0.378	0.576	0.765	0.576	0.378	0.185	0.185	0.765	0.378	0.185	0.765	0.378	8.009
**C4**	0.100	0.100	0.676	0.743	0.676	0.378	0.185	0.576	0.576	0.765	0.576	0.378	0.378	0.576	0.576	0.378	0.765	0.185	8.587
**C5**	0.500	0.100	0.500	0.100	0.743	0.185	0.576	0.185	0.185	0.576	0.576	0.576	0.185	0.185	0.378	0.576	0.378	0.765	7.272
**C6**	0.100	0.300	0.100	0.100	0.100	0.000	0.185	0.576	0.765	0.765	0.576	0.378	0.576	0.765	0.378	0.576	0.576	0.576	7.394
**C7**	0.500	0.100	0.300	0.100	0.500	0.378	0.000	0.185	0.185	0.576	0.378	0.576	0.185	0.576	0.185	0.576	0.576	0.185	6.065
**C8**	0.500	0.300	0.676	0.100	0.500	0.765	0.185	0.000	0.576	0.378	0.185	0.378	0.378	0.765	0.185	0.185	0.576	0.765	7.397
**C9**	0.100	0.300	0.100	0.500	0.500	0.378	0.576	0.185	0.000	0.576	0.378	0.765	0.576	0.576	0.185	0.185	0.576	0.378	6.836
**C10**	0.100	0.676	0.676	0.500	0.100	0.185	0.576	0.185	0.576	0.000	0.576	0.576	0.185	0.378	0.576	0.765	0.765	0.185	7.582
**C11**	0.100	0.100	0.676	0.500	0.676	0.765	0.378	0.185	0.576	0.378	0.000	0.185	0.576	0.765	0.765	0.765	0.576	0.765	8.730
**C12**	0.100	0.500	0.500	0.676	0.500	0.576	0.765	0.378	0.765	0.185	0.378	0.000	0.765	0.576	0.765	0.576	0.378	0.378	8.760
**C13**	0.500	0.676	0.100	0.100	0.676	0.378	0.378	0.765	0.576	0.765	0.765	0.576	0.000	0.378	0.378	0.378	0.765	0.185	8.336
**C14**	0.676	0.500	0.676	0.300	0.500	0.765	0.185	0.576	0.378	0.576	0.765	0.185	0.378	0.000	0.185	0.378	0.378	0.378	7.778
**C15**	0.100	0.100	0.500	0.100	0.676	0.378	0.576	0.185	0.185	0.185	0.378	0.576	0.185	0.378	0.000	0.576	0.576	0.765	6.421
**C16**	0.300	0.500	0.500	0.500	0.500	0.378	0.185	0.185	0.576	0.576	0.378	0.576	0.765	0.576	0.765	0.000	0.378	0.378	8.017
**C17**	0.100	0.500	0.100	0.300	0.676	0.378	0.185	0.378	0.378	0.185	0.185	0.765	0.765	0.378	0.765	0.576	0.000	0.576	7.189
**C18**	0.676	0.500	0.300	0.300	0.300	0.185	0.378	0.765	0.765	0.378	0.378	0.576	0.765	0.378	0.378	0.576	0.576	0.000	8.173

**Table 3:** Total relation matrix.

Challenges	C1	C2	C3	C4	C5	C6	C7	C8	C9	C10	C11	C12	C13	C14	C15	C16	C17	C18	Ri
C1	0.342	0.426	0.378	0.275	0.443	0.353	0.413	0.372	0.486	0.391	0.361	0.470	0.429	0.446	0.477	0.459	0.520	0.425	**1.864**
C2	0.191	0.349	0.280	0.265	0.375	0.275	0.291	0.254	0.325	0.326	0.285	0.374	0.299	0.350	0.391	0.371	0.433	0.298	**1.460**
C3	0.262	0.396	0.400	0.252	0.376	0.369	0.334	0.345	0.451	0.402	0.352	0.384	0.350	0.455	0.396	0.379	0.500	0.370	**1.686**
C4	0.259	0.353	0.431	0.351	0.482	0.377	0.341	0.370	0.465	0.457	0.408	0.438	0.401	0.468	0.453	0.434	0.538	0.383	**1.875**
C5	0.275	0.305	0.356	0.235	0.427	0.301	0.343	0.282	0.365	0.377	0.354	0.403	0.331	0.363	0.377	0.403	0.427	0.392	**1.598**
C6	0.228	0.334	0.311	0.243	0.360	0.289	0.297	0.327	0.429	0.402	0.359	0.386	0.378	0.430	0.377	0.402	0.453	0.371	**1.476**
C7	0.238	0.262	0.286	0.203	0.346	0.281	0.234	0.242	0.312	0.328	0.289	0.349	0.284	0.352	0.306	0.348	0.387	0.281	**1.335**
C8	0.278	0.337	0.373	0.237	0.400	0.369	0.300	0.272	0.414	0.363	0.318	0.386	0.357	0.431	0.357	0.361	0.455	0.394	**1.625**
C9	0.213	0.309	0.291	0.271	0.383	0.308	0.321	0.268	0.322	0.362	0.320	0.402	0.353	0.385	0.336	0.340	0.426	0.327	**1.466**
C10	0.224	0.376	0.380	0.292	0.367	0.313	0.342	0.286	0.411	0.324	0.359	0.411	0.338	0.397	0.408	0.426	0.479	0.333	**1.640**
C11	0.267	0.358	0.426	0.320	0.482	0.417	0.363	0.334	0.468	0.420	0.350	0.422	0.428	0.489	0.472	0.478	0.519	0.446	**1.852**
C12	0.262	0.397	0.402	0.339	0.463	0.395	0.403	0.349	0.481	0.398	0.387	0.399	0.442	0.467	0.469	0.454	0.497	0.400	**1.864**
C13	0.295	0.406	0.345	0.267	0.465	0.360	0.353	0.372	0.446	0.435	0.408	0.447	0.348	0.428	0.420	0.423	0.517	0.370	**1.779**
C14	0.305	0.374	0.394	0.273	0.421	0.386	0.316	0.345	0.413	0.403	0.394	0.383	0.372	0.372	0.380	0.402	0.459	0.373	**1.767**
C15	0.204	0.270	0.318	0.209	0.379	0.291	0.305	0.253	0.325	0.300	0.299	0.362	0.296	0.345	0.294	0.360	0.401	0.354	**1.380**
C16	0.266	0.379	0.381	0.304	0.434	0.350	0.324	0.309	0.437	0.410	0.364	0.434	0.417	0.437	0.447	0.368	0.467	0.376	**1.763**
C17	0.222	0.344	0.301	0.256	0.418	0.318	0.293	0.299	0.376	0.332	0.310	0.418	0.386	0.377	0.410	0.393	0.379	0.364	**1.541**
C18	0.316	0.387	0.362	0.286	0.421	0.338	0.350	0.375	0.465	0.393	0.365	0.444	0.429	0.426	0.415	0.435	0.495	0.342	**1.772**
**Ci**	**1.328**	**1.829**	**1.845**	**1.378**	**2.102**	**1.675**	**1.721**	**1.623**	**2.093**	**1.953**	**1.760**	**2.069**	**1.811**	**2.083**	**2.095**	**2.046**	**2.418**	**1.868**	
**Threshold value (**α**)** =	**0.366**																		
